# The Importance of Long-Term Video Electroencephalography Monitoring in the Differential Diagnosis of Epilepsy in Children

**DOI:** 10.7759/cureus.25700

**Published:** 2022-06-06

**Authors:** Mahmut Aslan, Serdal Gungor

**Affiliations:** 1 Pediatric Neurology, Mersin City Hospital, Mersin, TUR; 2 Department of Pediatric Neurology, Inonu University, Malatya, TUR

**Keywords:** treatment, diagnosis, non-epileptic paroxysmal event, epilepsy, video-eeg monitoring

## Abstract

Objective: Epilepsy is a condition in which abnormal, recurring, and excessive neuronal discharges caused by many different disorders in the central nervous system are observed. The rate of detecting epileptiform activity in the first interictal electroencephalography (EEG) of patients with epilepsy is around 40%, even when routine activation methods are applied. Video recordings simultaneously with EEG recordings enable the establishment of correlations between abnormalities.

Patients and methods: The long-term video-EEG monitoring (VEM) reports and images of 87 patients hospitalized in the video EEG service in the Pediatric Neurology Clinic of İnönü University between 2016 and 2020 were retrospectively analyzed in this study. Demographic information of the patients, such as age, gender, diagnosis/history, and length of hospital stay, was analyzed.

Results: A total of 87 patients were included in the study. Of the patients, 54 (62.1%) were male and 33 (37.9%) were female. The mean age was 107.57 ± 64.40 months. While 10 (11.5%) patients followed up with non-epileptic paroxysmal events (NEPE) were taking antiepileptic drugs (AEDs), AED treatments were discontinued after VEM. The diagnosis of 18 (20.69%) patients changed after VEM. The medications of 27 (31%) patients who took AEDs before VEM were discontinued after VEM. During VEM, sleep movement, psychogenic seizures, infantile masturbation, and tic disorder were observed in 19 (41.3%), 17 (36.9%), three (6.5%), and three (6.5%) patients, respectively.

Conclusion: In conclusion, VEM is the gold standard diagnostic method used to distinguish between epilepsy and NEPEs. After VEM, the clinical diagnosis of patients can change, and unnecessary drug administration may be prevented. Psychogenic nonepileptic seizures and NEPEs are common in the pediatric population; however, they are often overlooked.

## Introduction

Epilepsy is a condition in which abnormal, recurring, and excessive neuronal discharges caused by many different disorders in the central nervous system are observed [1]. Electroencephalography (EEG) is an easy and inexpensive method for diagnosing, supporting, classifying epilepsy, investigating the place of seizure onset, and monitoring patients [2]. However, EEG findings alone are not enough for the diagnosis of epilepsy [2]. The rate of detecting epileptiform activity in the first interictal EEGs of patients with epilepsy is around 40%, even when routine activation methods are applied [3]. However, the epileptiform discharge detection rate increases to 90% when the number of EEGs performed is 3-4 [4]. Therefore, 24-hour follow-up of some patients with long-term video-EEG monitoring (VEM) is required [5]. Video recording simultaneously with EEG recordings enabled the establishment of correlations between abnormalities [5]. Non-epileptic paroxysmal events (NEPEs) are paroxysmal attacks resembling an epileptic seizure but not due to abnormal cortical discharges [6]. Psychogenic NEPE constitutes approximately 35% of the non-epileptic cohort [6]. Today, long-term video-EEG monitoring is widely used for the diagnosis and classification of epilepsy, grading of the disorder, determination of focal location, electroclinical correlation, and pre-surgical examination [7]. Discontinuation of antiepileptic drug (AED) therapy is a more common method to identify seizures in children to reduce the time and cost associated with v-EEG. This study aims to present the experience of the only pediatric neurology clinic in the eastern region of the study country with the v-EEG monitoring method.

## Materials and methods

The VEM reports and images of 87 patients hospitalized in the Video EEG service in the Pediatric Neurology Clinic of İnönü University between 2016 and 2020 were retrospectively analyzed in this study. A detailed history was obtained from all patients, and neurological examinations were performed before hospitalization. Electroencephalography data were digitally recorded using a Micromed 32-channel EEG device. Scalp electrodes were placed according to the 10-20 system and fixed with collodion during the VEM procedure. A total of 87 patients were included in the study, with at least 12 hours of recording. Antiepileptic drug discontinuation was routinely applied to hospitalized patients. NEPE was identified and classified. Epileptic seizures were classified according to the International League Against Epilepsy (ILAE) 2017 criteria [8].

Demographic information of the patients, such as age, gender, diagnosis/history, and length of hospital stay, was analyzed. The number of seizures, the number of times the emergency button was pressed, and the compatible type of seizure in the event of a seizure were identified. Non-epileptic events were classified. Data such as ictal and interictal EEG pathology, sleep/awake EEGs, hospitalization and discharge diagnosis, and starting/discontinuation of an anti-epileptic drug (AED) were recorded and analyzed.

The study data were analyzed using the Statistical Package for Social Science (SPSS) version 15.0 software (IBM Corp., Armonk, NY). The chi-square test and the Mann-Whitney U test were used for nominal (categorical) outcome variables.

An ethics committee approval was received for our study by İnönü University Scientific Research and Publication Ethics Committee (Malatya/Turkey) with the decision number 2019/416 in 2019. The information and documents of the patients in our study are available in the database of the İnönü University Faculty of Medicine. It can be accessed from the database if necessary.

## Results

A total of 87 patients were included in the study. Of the patients, 54 (62.1%) were male and 33 (37.9%) were female. The age range of the patients was 15 days to 18 years, and the mean age was 107.57 ± 64.40 months. The duration of hospitalization in the video-EEG service of the patients was 32.55 ± 12.26 hours. The patients took 1.35 ± 1.21 unit AEDs during the hospital stay. The patient pressed the emergency button 546 times, and the mean number of pressing the emergency button was 6.27 ± 11.03. They pressed the button 191 times (34.98%) for seizures and 355 (65.02%) for NEPEs (Table 1).

**Table 1:** Demographic and video-electroencephalography monitoring stay characteristics of the patients VEM: video electroencephalography monitoring, NEPE: non-epileptic paroxysmal events, AED: antiepileptic drug

Features	Values
Age range (months)	15 days to 18 years
Mean age (months)	107.57±64.40
Female (n/%)	33 (37.9)
Male (n/%)	54 (62.1)
VEM stay (hours/mean)	32.55±12.26
Number of pressing emergency button (mean)	546 (6.27±11.03)
Seizure	191 (35%)
NEPE	355 (65%)
AED use (mean)	1.35±1.21

Among the patients included in the study, neurological examination was normal in 69 (79.32%) patients and abnormal in 18 (20.68%) patients. Magnetic resonance imaging (MRI) of 54 (62.1%) patients was normal. Fifteen (17.2%) patients had abnormal MRI, and 18 (20.7%) patients had no MRI. Ictal epileptic abnormalities were observed in 19 (21.8%) patients. Eight (9.2%) of these abnormalities were focal epileptic abnormalities, nine (10.3%) were generalized epileptic abnormalities, and two (2.3%) were epileptic spasms. Ictal and interictal epileptic abnormalities were not observed in 46 (52.9%) of the patients. Interictal epileptic abnormality was observed in 14 (16.1%) patients, but the ictal recording was not obtained. Of the interictal epileptic abnormalities, four were generalized, four were focal, three were multifocal, and three were secondary generalized. Seizures and NEPE were concurrently observed together in eight (9.2%) patients (Table 2).

**Table 2:** Epileptic anomalies in video-electroencephalography monitoring NEPE: non-epileptic paroxysmal events, CP: cerebral palsy

Diagnosis	Pre-VEM n/%	Post-VEM n/%
Primary epilepsy	12/13.8	11/12.6
NEPE	30/34.5	46/52.9
Primary epilepsy + NEPE	29/33.3	14/16.1
CP + epilepsy	8/9.2	8/9.2
Autism + epilepsy	3/3.4	3/3.4
Mental retardation + epilepsy	3/3.4	3/3.4
Dravet syndrome + epilepsy	2/2.3	2/2.3

Twelve (13.8%) patients, 30 (34.5%) patients, 29 (33.3%) patients, eight (9.2%) patients were followed up with primary epilepsy, NEPE, primary epilepsy + NEPE, and cerebral palsy (CP) + epilepsy, respectively, before video EEG. During the video EEG, frontal epilepsy was observed in one of 30 patients followed up with NEPE. The other 29 patients were followed up with NEPE. In two patients who were followed up with primary epilepsy, it was observed that they did not have epilepsy but had NEPE, and therefore AED was discontinued. In 15 patients followed up with primary epilepsy + NEPO, it was observed that they did not actually have epilepsy and had NEPO and AED treatments discontinued (Table 3). While 10 (11.5%) patients who followed up with NEPE were taking AEDs, AED treatments were discontinued after VEM. The diagnosis of 18 (20.69%) patients changed after VEM. The medications of 27 (31%) patients who took AEDs before VEM were discontinued after VEM.

**Table 3:** Pre-video-electroencephalography monitoring and post-video-electroencephalography monitoring diagnoses NEPE: non-epileptic paroxysmal events, EA: epileptic abnormality

Epileptic anomalies	n	%
Presence of ictal EA (type of seizure)	19	21.8
Focal motor	6	6.1
Focal non-motor	2	3.1
Generalised motor	7	8.1
Generalised non-motor	2	2.2
Epilptic spasm	2	2.2
No interictal + Ictal EA	46	52.9
Presence of interictal EA, no ictal EA	14	16.1
Primary generalised	4	4.6
Focal	4	4.6
Multifocal	3	3.45
Sekondary generalised	3	3.45
Seizure + NEPE	8	9.2

During VEM, sleep movement, psychogenic seizures, infantile masturbation, and tic disorder were observed in 19 (41.3%), 17 (36.9%), three (6.5%), and three (6.5%) patients, respectively. Fejerman syndrome, hemifacial spasm, central hypoventilation syndrome, and tonic upward gaze were observed in one (2.17%) patient.

## Discussion

Video-EEG monitoring is an important diagnostic method in patients with epilepsy for distinguishing between seizures and NEPEs [6,9]. While the rate of detecting epileptiform abnormalities in the first routine EEG performed after a seizure is 29-55%, it can increase to 39-72% in the third EEG [10]. The epileptiform abnormality detection rate in an ambulatory EEG recording of 72-96 hours in patients with recurrent paroxysmal events was found to be 68%, which was similar to the third routine EEG. As a result, the sensitivity of long-term EEG is similar to that of repeated routine EEG. While the rate of detecting interictal epileptic abnormalities with VEM is 30-40% in the literature, interictal EA and ictal EA of 14.9% and 20.7%, respectively, were identified in the present study [11]. In previous studies, the mean recording time was three to four days in adults and one to two days in children [12]. In the present study, the mean recording time was 32.55 hours.

Epileptic seizure and NEPE are among the clinical conditions in which diagnostic confusion occurs. Misdiagnosis leads to long-term use of wrong and unnecessary drugs, drug side effects, additional financial burden, delays in the recovery period of patients, and thus social problems [13]. NEPE was found to be between 15% and 33% in previous VEM studies. One of the most common problems encountered in epilepsy centers is NEPE [14]. In some patients, epilepsy and NEPE may coexist [15]. The association of epilepsy + NEPE has been reported to be between 5% and 50% in various studies [16]. In the present study, while 34.5% of patients had NEPE and 33.3% had primary epilepsy+NEPE association before VEM, it was identified that 52.9% of the patients had NEPE and 16.1% of patients had primary epilepsy+NEPE after VEM.

In some comprehensive studies conducted previously, a change at a rate of 52.3-53% was observed in diagnosis [17]. In another study, the diagnosis changed in 18% of the patients, and AEDs were discontinued in 30% of the patients [18]. In the present study, the diagnosis of 20.69% of the patients changed after VEM, and AEDs were discontinued for 31% of the patients. Psychogenic nonepileptic seizures represent 20-30% of patients with drug-resistant epilepsy. In similar studies, sleep movements constituted 40-70% of NEPEs [16]. In the present study, 36.9% of NEPEs were psychogenic and 41.3% were sleep movements.

The present study confirms the larger-scale results of previous studies on the use of v-EEG for the non-emergency and non-surgical assessment of events. Moreover, in accordance with the literature, it supports the data that the diagnosis of primary epilepsy can be mistaken for NEPEs. In patients who may have a normal interictal EEG pattern, such as Dravet's syndrome, but have frequent seizures described during the day or in patients who have intertwined paroxysmal events, such as cerebral palsy and autism and epilepsy, v-EEG is a highly effective and non-invasive method in the management of these patients [19,20]. In the present study, there was no change in the diagnosis after v-EEG in patients with Dravet syndrome, autism, and cerebral palsy. On the other hand, it is considered that v-EEG is the method of first choice in patients with the diagnosis of primary epilepsy after v-EEG and in patients with this diagnosis and inadequate response after antiepileptic treatment.

There are some limitations to the present study. The single-centered study approach was an important limitation. In addition, the study included retrospective data. It should be noted that more studies and larger studies involving more clinics are needed to better classify PNES and NEPEs and facilitate diagnosis and treatment in children.

## Conclusions

In conclusion, VEM is the gold standard diagnostic method used to distinguish between epilepsy and NEPEs. After VEM, the clinical diagnosis of patients can change, and unnecessary drug administration may be prevented. Psychogenic nonepileptic seizures and NEPEs are common in the pediatric population; however, they are often overlooked. Both conditions are substantially misdiagnosed as epilepsy and AEDs are started. Diagnosis and differentiation of PNESs from actual epileptic seizures became easier. v-EEG monitoring is of particular importance in terms of medicine and law. Systematic and uniform classification of childhood NEPE cases may help to improve standardization. A wider range of uses is essential for early detection and easy comparison. Diagnosis and treatment cannot exclude a detailed assessment of underlying psychological stressors and their associated comorbidities. The present study aimed to present a practical approach to clinicians faced with complex conditions such as NEPE in the pediatric age group. The aim was also to raise awareness of diagnosis and, above all, differential diagnosis with other neuropsychiatric diseases, especially epilepsy. To the best of our knowledge, the present study is essential as it is the most comprehensive study done in the eastern region of Turkey according to the current literature.
